# Management of Forgotten Ureteral Stents

**DOI:** 10.5812/numonthly.9369

**Published:** 2013-03-30

**Authors:** Mustafa Burak Hoşcan, Ahmet Tunçkıran

**Affiliations:** 1 Alanya Practice and Research Center, Department of Urology, Faculty of Medicine, Başkent University, Alanya, Turkey

**Keywords:** Disease Management, Prevention and Control, Stents, Ureter

Dear Editor,

We read with great interest the study reported by Rabani (1). The author reported their experiences in endoscopic management of forgotten ureteral stents with large burden stone encrustation. As the author stated, ureteral stents are widely used in urology practices. However, complications including migration, fragmentation, and encrustation can be related to stents and sometimes the stents may possibly be forgotten. We would like to add some additional comments and suggestions on the issue of forgotten ureteral stents.

Although ureteral stents have various advantages, forgotten stents are a challenging situation even for the experienced endourologists. Forgotten stents can result in serious morbidity, mortality, and an increased financial burden for health services (2). Forgotten ureteral stents usually arise from poor compliance of the patient or failure of the physician to adequately counsel the patient. One important medicolegal issue also needs to be emphasized. The attending urologist is ultimately responsible for the removal of stent and also responsible for the complications if the patient with stent is lost to follow-up.

Gravas et al. (3) underscored some important points in the management of forgotten ureteral stents. They reported that a thorough, preoperative, imaging evaluation is necessary to decide the treatment strategy, which should aim to keep the number of necessary interventions as low as possible. They also stated that the most controversial and difficult part in the management of forgotten ureteral stents is the encrusted upper curl, and the critical question is which method out of percutaneous nephrolithotomy, ureteroscopy and extracorporeal shockwave lithotripsy is the most appropriate.

As Rabani (1) mentioned, while endourological manage¬ment of these stents achieves success in majority of cases with minimal complications, the best treatment would be prevention of this complication. For achieving this important goal, some investigators have offered different strategies. Lynch et al. have suggested an electronic stent extraction reminder facility recall base to prevent forgot¬ten stents (4). Sancaktutar et al. Described and presented the initial results of a computer-based system that tracks ureteral stents and automatically sends a reminder through a short message service to both the patient’s and the urologist’s mobile phones (2). Recently, Chew et al. even described a novel biodegradable ureteral stent in a porcine model (5). 

Although endourologic management of forgotten ureteral stents possible in most of the cases with success, further efforts and studies must be made for prevention strategies and possibilities for new kind or type of ureteral stents.
